# Coagulation Profile of the Healthy Miranda’s Donkey

**DOI:** 10.3390/ani14142031

**Published:** 2024-07-10

**Authors:** Grasiene Silva, Felisbina L. Queiroga, Zélia Cruz, Amana Maia, Ana C. Silvestre-Ferreira

**Affiliations:** 1 Departamento de Ciências Veterinárias, Universidade de Trás-os-Montes e Alto Douro (UTAD), 5000-801 Vila Real, Portugal; grasivet@hotmail.com (G.S.); fqueirog@utad.pt (F.L.Q.); amanamedvet@gmail.com (A.M.); 2 Centro de Ciência Animal e Veterinária (CECAV), Universidade de Trás-os-Montes e Alto Douro (UTAD), 5000-801 Vila Real, Portugal; 3 Laboratório Associado para a Ciência Animal e Veterinária–AL4AnimalS, 1300-477 Lisboa, Portugal; 4 Centre for the Research and Technology of Agro-Environmental and Biological Sciences (CITAB), 5000-801 Vila Real, Portugal; 5 AEPGA-Associação para o Estudo e Proteção do Gado Asinino, Largo da Igreja, n° 48, 5225-011 Atenor, Portugal; zeliacruz.aepga@gmail.com

**Keywords:** coagulation, Miranda’s donkey, reference intervals, Start^®^ 4-Diagnostica-Stago

## Abstract

**Simple Summary:**

Miranda’s donkey is an autochthonous Portuguese breed that is important for the culture and economy of the country’s northern region. Knowledge of the physiological characteristics of the breed is crucial for its preservation, and even if studies have been conducted, none have focused on its coagulation profile. The aim of this study was to establish reference intervals for coagulation and assess the influence of sex and age. For this, seven parameters involved in coagulation were analyzed in blood samples from 75 clinically healthy animals. Statistical differences were only found between ages in two parameters, platelet count and plateletcrit, both of which were higher in young animals. The reference intervals described here can help monitor health and guide the diagnosis and treatment of sick Miranda’s donkey, contributing to its preservation.

**Abstract:**

Miranda’s donkey, originating in northern Portugal, is an autochthonous breed that is deeply intertwined with the region’s culture and economy. Knowledge of the physiological characteristics of the breed is important for its preservation, and several studies have been carried out, but none have focused on its coagulation profile. The aim of this study was to establish reference intervals (RIs) for coagulation in healthy Miranda’s donkey and to assess the influence of sex and age. Blood samples from 75 clinically healthy animals were analyzed for seven coagulation parameters: four using IDEXX ProCyte Dx and three using Start^®^ 4-Diagnostica-Stago. The RI values were calculated following the ASVCP guidelines and with the Reference Advisor V.2.1 software. To analyze the influence of sex and age, SPSS version 29 was used. No significant differences were found between sexes (*p* > 0.05), but statistically significant differences were found between ages (*p* < 0.05) for platelet count and plateletcrit (both higher in young animals). The RIs described here can help monitor health and guide the diagnosis and treatment of diseased Miranda’s donkeys, contributing to their preservation. Our study encourages further research on coagulation in donkeys and the use of different methodologies to obtain information for veterinarians working with this species.

## 1. Introduction

Miranda’s donkey is a breed originating from Portugal, more specifically from the region of Miranda do Douro in the north of Portugal [1]. This breed is known for its resistance, robustness, and adaptability to the adverse climatic conditions of the region, as well as for its physical and behavioral characteristics, making it suitable for traction work and agricultural transport [1,2].

However, like many other breeds of working animals, Miranda’s donkey faces challenges due to the modernization of agriculture and technological advances, which have resulted in a decline in its population over the years and its classification as an endangered breed [3,4,5].

To preserve the breed, conservation efforts and programs to encourage reproduction have been implemented, such as the use of these animals for tourism and cultural activities, the production of cosmetics based on donkey milk, and donkey therapy [6,7]. Consequently, there is an increasing need to understand the physiological characteristics of this breed to maintain its health. Currently, research has been conducted on Miranda’s donkey in several areas, including reproduction, dentistry, nutrition, parasitology, hematology, and serum biochemistry [8,9,10,11,12,13].

However, some issues within the breed remain unresolved, such as the reference intervals (RIs) for coagulation tests. Knowledge of these data is fundamental because the coagulation profile is essential in several clinical conditions, from simple castration to serious diseases that result in coagulation disorders or, less frequently, hereditary diseases [14,15,16]. Disseminated intravascular coagulation (DIC), an acquired coagulopathy characterized by hyperactivation of the coagulation system with the consumption of platelets, clotting factors, and coagulation inhibitors, is a major blood disorder. DIC occurs secondary to a disease, for example, in horses with gastrointestinal diseases, ischemic/inflammatory colic, colitis, volvulus of the greater colon, and sepsis [17,18,19]. Depending on its severity, DIC may present in a subclinical or clinical form. The development of DIC is directly related to disease prognosis. Therefore, a coagulation panel and/or global assessment of hemostasis is recommended at the admission/first visit and serially during the hospitalization of any equine patient presenting gastrointestinal disease and/or sepsis [20].

Generally, clinical practice involving donkeys represents a challenge for veterinarians, as certain issues have been extensively studied in horses but not in donkeys. Consequently, many of the clinical conditions observed in donkeys are treated based on recommendations for horses. For example, hemostasis in donkeys remains poorly understood, and research indicates that determining a donkey’s parameters based on a horse’s RIs might be inadequate [21,22]. Despite their similarities, there are numerous differences among these species [23,24].

Other factors that can influence coagulation, which should also be studied in donkeys, are the interference of age, sex, and the reproductive status of females. Studies in horses have indicated that the number of platelets may be higher in young animals and females at the end of gestation [22]. Pregnant females may also exhibit increased fibrinogen levels near and after parturition [25].

The aim of our study was to establish the RIs for coagulation parameters in Miranda’s donkey, to evaluate the possible influence of sex, age, or pregnancy, and to compare the results with those previously described in equids.

## 2. Materials and Methods

### 2.1. Animals

Seventy-seven clinically healthy Miranda donkeys, resident in northern Portugal (41°42′ N, 06°48′ W, 652 m altitude) and registered in the Association for the Study and Protection of Donkey Cattle (AEPGA), participated in this investigation. These animals were periodically monitored by AEPGA veterinarians and subjected to prophylactic anthelmintic treatment [26]. Inclusion criteria to participate in the study were to be considered healthy in clinical examination, with no signs of disease, or treatments in the last six months.

Blood sampling was carried out during routine tests at AEPGA. This study was previously approved by the Ethics Committee for Animal Welfare (ORBEA) of the University of Trás-os-Montes and Alto Douro (UTAD)—i467-e-CECAV-2022. The owners or caretakers stated the animals’ health status and gave written informed consent for the use of data and the remaining blood samples. Pregnant and lactating mares were also included [27,28].

To investigate the effect of sex, the donkeys were divided into two groups: males (33 animals) and females (42 animals); of these, 12 were pregnant females. Regarding the effect of age on the coagulation profile, donkeys were divided into two groups: 20 young (1–3 years) and 55 adults (≥4 years) [27,28].

### 2.2. Animals Sample Collection and Analysis

Samples were collected in April during the breed’s annual prophylactic program through the morning period. Blood collection was performed by puncture of the jugular vein using a 21-gauge needle (BD Vacutainer PrecisionGlide, Plymouth, UK), into 4 mL blood tubes with K_3_EDTA (BD Vacutainer), and 4 mL blood tubes with citrate 3.2% (BD Vacutainer) at a blood/citrate ratio of 9:1. At the time of collection, veterinarians performed anamnesis and physical examination in order to classify the animals as clinically healthy together with hemogram and serum biochemistry information.

Blood samples in K_3_EDTA were homogenized and processed for platelet count (PLT), mean platelet volume (MPV), platelet distribution width (PDW), and plateletcrit (PCT), in a laboratory set up on AEPGA’s premises, using the IDEXX ProCyte Dx, automatic hematology analyzer, previously validated for the species, according to the manufacturer’s recommendations, within 4 h from collection [29].

The blood samples collected in citrate tubes were subjected to routine centrifugation (800× *g* for 5 min), and the plasma obtained was refrigerated and transferred to identified aliquots and sent for analysis to the Veterinary Clinical Pathology Laboratory at UTAD. Subsequently, fibrinogen (Fb), prothrombin time (PT), and activated partial thromboplastin time (aPTT) were evaluated using semi-automatic equipment (Start^®^ 4-Diagnostica-Stago, Asnières sur Seine, France); for this purpose, plasma samples were thawed to reached a temperature of 37 °C and analyzed following calibration and quality control standards as indicated by the manufacturer’s instructions.

### 2.3. Statistical Analyses

The analysis of mean, median, minimum–maximum, RI, confidence interval (CI), and 90% CI for the lower and upper limits was carried out using Reference Values Advisor 2.1 application [30]. This software uses the Anderson–Darling test to assess normality. To detect possible outliers, Tukey and Dixon–Reed methods were used [30]. Reference intervals were determined according to the guidelines of the American Society of Veterinary Clinical Pathology (ASVCP) [31].

To investigate possible significant differences in sex and age in the coagulation profile, analyses were performed using the SPSS software version 29. The normality of the samples was tested using the Shapiro–Wilk and Kolmogorov–Smirnov tests. Variables with Gaussian distribution were subjected to analysis of variance (ANOVA), and non-Gaussian variables were subjected to the Kruskal–Wallis test [32]. Differences were considered statistically significant at *p* < 0.05.

Animals that presented discrepant results and were considered possible outliers were excluded from the statistical analysis.

## 3. Results

Seventy-five healthy Miranda’s donkey were included in the study, consisting of 42 females with a mean age of 8.0 ± 6.1 years (range 1 to 25 years) and 33 males with a mean age of 8.3 ± 5.1 years (range 1 to 18 years). Seven parameters were measured to evaluate coagulation efficiency. All data are presented as mean, median, standard deviation (SD), minimum and maximum values (min–max), RI, 90% confidence interval (CI) for the lower reference limit (LRL), and 90% CI for the upper reference limit (URL). The RI values for all the animals are described in Table 1.

The aPTT, Fb, and PLT followed a normal distribution. In contrast, PT, MPV, PDW and PCT did not follow the normal distribution model. Three results for aPTT, one for PT, and four for fibrinogen were considered outliers and removed from the statistical analysis.

No significant differences were found between sexes for any of the parameters analyzed (*p* > 0.05). Twelve of the forty-two females in our study were pregnant at different gestation periods. Therefore, analyses were also conducted to identify the influence of pregnancy; however, no significant differences were observed (*p* > 0.05). The mean values of all parameters for each pregnant female are listed in Table 2.

The results for the 55 adult and 20 young patients, including RI, are presented in Table 3. For the young group, it was not possible to calculate the LRL or URL for aPTT/s and Fb, as they were not computable by the system. Regarding age, differences were found for PLT and PCT levels with a higher average in young patients (*p* < 0.05) (Table 4).

## 4. Discussion

Studies of RI values for blood-clotting tests in donkeys are limited. Consequently, veterinarians use established RIs for horses to assess the health of donkeys. However, this practice can lead to erroneous interpretations of the health status of these animals, owing to differences between species [21,23,24]. The present study is the first to determine the RIs for coagulation in Miranda’s donkey, using the simplest and most frequently used coagulation tests in the clinic.

Hemostasis encompasses all physiological mechanisms aimed at retaining blood within the vessels and in a fluid state. Normal hemostasis has three components: primary hemostasis, secondary hemostasis, and fibrinolysis [33]. Platelets are the basis of primary hemostasis, which is a mechanism that provides plugs to repair small vascular defects through their interaction with activated endothelium or subendothelial collagen [20,34].

To ensure efficient hemostasis, a minimum number of platelets is required, which must function correctly. In our study, the average number of PLT in all the animals, regardless of sex and age, was higher than that reported from horses of different breeds [21,22,35,36] and donkey breeds, such as Andalusian [21,22], Herzegovinian [37], and mixed breeds [38,39]. PLT might be influenced by factors such as age, sex, breed, reproductive status, or exercise and training [34,40].

No significant differences were found in PLT, MCV, PDW, or PCT in relation to sex in our study (*p* > 0.05). In contrast, significant differences were found in relation to age for PLT and PCT (*p* < 0.05) with higher values in young animals than in adult animals. The highest number of PLT in young individuals has also been described in other European donkey breeds, such as the Spanish Catalan breed, the French Normandi and Contentin breeds, and the Serbian Balkan breed [41,42,43]. The decrease in PLT is possibly a consequence of the decrease in platelet production with age [44]. PCT level, which is directly related to PLT, was predicted to be higher in young animals.

No statistically significant differences were found in PLT, MCV, PDW, and PCT levels related to pregnancy. The females in our study were at different gestation periods, and platelet counts were reduced in females at the end of pregnancy compared to females in the early stages of gestation. It was not possible to compare it with other animals of the same species, as studies carried out with donkeys involved only nonpregnant females [21,22]. In horses, this influence remains controversial because some studies conducted on pregnant mares have not found a significant effect on platelet parameters [45,46]. However, previous studies described a progressive decrease in platelet count during pregnancy in the Carthusian breed. These authors justified this influence as a dynamic effect of the hormone during pregnancy combined with increased levels of thromboxane B2 produced by the placenta, chorion, and amnion [47,48].

The simplest and most practical tests used to evaluate secondary hemostasis are PT and aPTT, which evaluate the extrinsic, intrinsic, and/or common coagulation pathways. These screening tests use specific reagents to sequentially activate clotting factors in a process known as the clotting cascade [19,49]. These tests can be performed using semi-automated benchtop coagulometric analyzers or portable devices, which in some studies have demonstrated good agreement and precision compared to laboratory methods. Point-of-care equipment also allows faster results, as it can be used close to the animals without the need for sample transport [19]. The literature on the coagulation profile of donkeys is scarce. In our study, and others performed in donkeys, only semi-automatic analyzers were used [21,22].

In our study, Miranda’s donkey presented PT and aPTT values higher than those initially described in the horse and Spanish Andalusian breed donkey [21], but they were close to those later published in the same breed [22]. The tests in our study and those of Mendoza et al. [21] and Perez-Ecija and Mendoza [22] were performed using the same semi-automatic device, Start^®^ 4-Diagnostica-Stago.

No statistically significant differences were found in the aPTT and PT in relation to sex and age in our study. Our aPTT results differ from those reported in Andalusian donkeys, which showed significant differences between age groups and sex. Animals aged between 5 and 10 years had more prolonged aPTT, and females had shorter aPTT times than males [22]. No statistically significant differences were observed in terms of pregnancy. This result corroborates those described in pregnant mares that maintained constant aPTT and PT without pre-and postpartum influence [25].

A prolonged PT may indicate deficiencies in one or more of the following factors: V, VII, X, prothrombin (factor II) or Fb. These can be caused by liver problems or vitamin K deficiency, as factors II, VII, and X are dependent on this vitamin. Prolonged aPTT indicates deficiencies in one or more of the following factors: VIII, IX, XI and XII; prothrombin (factor II); or Fb [19,20,50]. In donkeys, there are no studies that report changes in TP, aPTT and coagulation factors in sick animals. Only one study reported changes in Fb values in animals with induced endotoxemia [51]. Contrarily, in horses, several studies report changes in PT, aPTT and coagulation factors in animals with colic [52], various gastrointestinal disorders [53], atrial fibrillation [54] and other disorders [50].

Fibrinogen is crucial in coagulation because after the activation of factors involved in the coagulation cascade and the activation of prothrombin in its active form, thrombin, soluble Fb is converted into insoluble fibrin. This fibrin binds to platelets to occlude blood flow to an injured vessel [55]. Deficiency in fibrin formation as well as excess production/deficiency in its elimination (deficiency in fibrinolysis) can cause serious problems with blood loss or the formation of free thrombi in the circulation [15,50].

Fibrinogen is also important in inflammatory processes, as it is a positive acute phase protein whose concentration increases during inflammation. In equines, inflammation can increase Fb values even in the absence of leukogram changes, and therefore, it should be included in the routine blood diagnosis of equines [56].

The results of our study for Fb were lower than those found in the Spanish Andalusian breed [21] but were close to those later published in the same breed [21,22]. Our results were also lower than those of previous studies carried out in horses [21,53,57]. The methodology used in the studies cited on donkeys and horses, heat precipitation, was different from that used in our study, with the exception of the study performed by Mendez-Angulo et al. [53], which also used semi-automatic equipment but from another manufacturer.

The heat precipitation method provides an estimate of the plasma concentration of fibrinogen, which is adequate for evaluating hyperfibrinogenemia but lacks the analytical sensitivity necessary for assessing hypofibrinogenemia [55]. Another limiting factor is the subjective interpretation, which depends on the accuracy of the observer. The semi-automatic method allows for less subjective evaluation by employing electromagnetic assessment within a controlled environment and temperature, with the device indicating the amount of fibrinogen. Although it is more accurate, it is important for the operator to follow the manufacturer’s instructions, calibrations, and controls.

No statistically significant differences were found in Fb related to age, sex, or pregnancy in our study. Another study found significant differences in Fb in pregnant mares, which increased at the end of pregnancy and reached its limit after parturition [25].

It is important to highlight that in order to reach a diagnosis of hemostatic disorder, it is necessary to jointly evaluate the animal’s history, anamnesis, possible clinical symptoms, blood count, and, when necessary, other complementary tests that may justify the results found in PLT, TP, aPTT, and Fb [20,55]. The technique used should also be considered, as different exams, techniques, and equipment may present different results. It is advisable that the laboratory be contacted to obtain information about the procedures for sample collection and transport in order to obtain results of maximum reliability [55].

Another important aspect is the care to be taken during the collection and performance of the tests. It is important that venipunctures are aseptic without tissue contamination, which could result in undesirable platelet aggregation. Blood collection is performed with the least possible stress for the animal in a vein that is easily accessible and without unnecessary trauma [55,58,59]. Suitable tubes have the correct anticoagulant (citrate) with respect to the anticoagulant/blood ratio (9:1), because compared to other tubes, citrate tubes contain a greater amount of anticoagulant. Therefore, it is essential that these tubes are filled to the appropriate level because, for example, if they are underfilled, the relative excess of anticoagulant in the sample can prolong the results of PT and aTTP [60]. In addition, care should be taken when polycythemia is present. Patients with a markedly increased hematocrit have less plasma, which can result in a relative excess of anticoagulant and increased PT and aTTP [60].

## 5. Conclusions

The Miranda’s donkey needs to be preserved, and for this it is necessary that the physiological aspects of the breed are known. The breed’s coagulation profile is important, and tests such as PLT, TP, aPTT, and Fb counts, are used together, to identify blood clotting abnormalities, assisting in both simple and complex procedures. In our study, observed results differ from those reported in horses and other breeds of donkeys. Therefore, the results described here are important to guide veterinarians.

New studies on coagulation should be carried out on Miranda’s donkey, using different techniques and, when possible, with a larger number of animals. We also reinforce the need for studies in other donkey breeds to assist in the treatment of these animals with reference intervals appropriate to the species.

## Figures and Tables

**Table 1:** Reference intervals for coagulation profile in healthy Miranda donkeys (*n* = 75).

Analyte/Units	*n*	Mean ± SD	Median	Min–Max	RI	LRL 90% CI	URL 90% CI
aPTT/s	72	49.6 ± 8.5	49.6	32.7–82.0	35.1–71.4	32.7–38.7	63.1–82.0
PT/s	74	15.6 ± 2.6	15.2	12.1–26.5	12.5–23.1	12.1–12.8	20.3–26.5
Fb (mL/dL)	71	213.0 ± 54.4	207.0	96.0–374.0	96.8–361.2	96.0–143.2	305.2–374.0
PLT (K/µL)	75	221.1 ± 56.0	228.0	74.0–344.0	93.8–341.3	74.0–139.0	298.3–344.0
MPV (fL)	75	6.1 ± 0.5	6.0	5.3–7.9	5.3–7.6	5.3–5.4	7.1–7.9
PDW (%)	75	6.8 ± 0.7	6.7	5.8–8.7	5.8–8.7	5.8–6.0	8.1–8.7
PCT (%)	75	0.11 ± 0.0	0.11	0.03–0.19	0.0–0.2	0.0–0.1	0.2–0.2

*n*: number; SD: standard deviation; Min: minimum; Max: maximum; RI: reference intervals; LRL: lower reference limit; URL: upper reference limit; CI: confidence interval; s: seconds; aPPT: activated partial thromboplastin time; PT: prothrombin time; Fb: fibrinogen; PLT: platelets; MPV: mean platelet volume; PDW: platelet distribution width; PCT: plateletcrit.

**Table 2:** Reference intervals for coagulation tests in pregnant female Miranda donkeys (*n* = 12).

Female	MP	aPTT/s	PT/s	Fb (mL/dL)	PLT (K/µL)	MPV (fL)	PDW (%)	PCT (%)
P1	1 month	49.9	15.6	215	267.00	5.8	6.5	0.13
P2	1 month	54.2	21.9	158	212.00	5.6	6.3	0.09
P3	1 month	56.4	22.6	187	294.00	5.8	6.3	0.14
P4	2 months	45.3	13.3	194	261.00	6.2	6.8	0.14
P5	2 months	43.6	13.2	374	196.00	6.0	6.6	0.10
P6	2 months	53.6	20.3	202	274.00	5.7	6.5	0.13
P7	3 months	42.9	14.3	229	164.00	6.7	7.4	0.09
P8	3 months	41.5	15.5	292	222.00	6.9	7.7	0.13
P9	9 months	44.0	15.2	181	233.00	6.3	7.0	0.12
P10	10 months	51.6	14.8	186	142.00	6.3	6.8	0.07
P11	12 months	41.4	13.1	228	214.00	5.8	6.3	0.09
P12	12 months	51.2	16.9	255	158.00	7.1	8.0	0.08

*n*: number; MP: month of pregnancy; aPPT/s: activated partial thromboplastin time; PT/s: prothrombin time; Fb: fibrinogen; PLT: platelets; PDW: platelet distribution width; PCT: plateletcrit; MPV: mean platelet volume; s: seconds.

**Table 3:** Reference intervals for coagulation tests in healthy adults and young Miranda donkeys.

Analyte/Units	*n*	Mean ± SD	Median	Min–Max	RI	LRL 90% CI	URL 90% CI
Adults							
aPTT/s	53	48.7 ± 7.2	48.3	32.7–69.1	33.7–66.7	32.7–38.3	59.6–69.1
PT/s	54	15.4 ± 2.5	15.2	12.1–26.5	12.3–24.8	12.1–12.7	18.3–26.5
Fb (mL/dL)	52	206.6 ± 58.0	198.5	96.0–374.0	96.3–368.8	96.0–131.8	301.0–374.0
PLT (K/µL)	55	206.4 ± 51.9	212.0	74.0–310.0	82.8–308.4	74.0–139.0	283.0–310.0
MPV (fL)	55	6.1 ± 0.6	5.9	5.3–7.9	5.3–7.8	5.3–5.4	7.1–7.9
PDW (%)	55	6.8 ± 0.7	6.6	5.8–8.7	5.9–8.7	5.8–6.1	8.1–8.7
PCT (%)	54	0.10 ± 0.0	0.10	0.03–0.15	0.0–0.1	0.0–0.1	0.1–0.2
Young							
aPTT/s	19	52.4 ± 10.9	50.1	39.0–82.0	37.1–92.3	*	*
PT/s	20	16.1 ± 2.8	15.4	12.9–22.6	12.0–25.4	11.5–12.8	20.5–32.9
Fb (mL/dL)	19	230.3 ± 39.5	230.0	170.0–306.0	160.3–331.4	*	*
PLT (K/µL)	20	261.7 ± 46.8	272.0	143.0–344.0	144.2–350.4	94.0–194.6	324.3–376.0
MPV (fL)	20	6.1 ± 0.5	6.1	5.3–7.1	5.1–7.2	4.9–5.4	6.8–7.6
PDW (%)	20	6.9 ± 0.7	6.7	5.8–8.6	5.8–8.7	5.5–6.0	7.8–9.7
PCT (%)	20	0.13 ± 0.0	0.13	0.11–0.17	0.1–0.2	0.1–0.1	0.2–0.2

*n*: number; SD: standard deviation; Min: minimum; Max: maximum; RI: reference intervals; LRL: lower reference limit; URL: upper reference limit; CI: confidence interval; s: seconds; aPPT: activated partial thromboplastin time; PT: prothrombin time; Fb: fibrinogen; PLT: platelets; MPV: mean platelet volume; PDW: platelet distribution width; PCT: plateletcrit; *: non-computable by the system.

**Table 4:** Effect of age on platelet parameters in Miranda donkeys.

Effect of Age			
Analyte/Units	Adults (*n* = 55)	Young (*n* = 20)	
	Mean ± SD	Mean ± SD	*p*-value
PLT (K/µL)	206.4 ± 51.9	261.7 ± 46.8	<0.001
	Median ± SD	Median ± SD	*p*-value
PCT (%)	0.10 ± 0.0	0.13 ± 0.0	<0.001

*n*, number; PLT, platelet; PCT, plateletcrit; SD, standard deviation.

## Data Availability

All the data supporting the results are included in the manuscript. The dataset is available from the corresponding author on reasonable request.
